# Impact of mhealth messages and environmental cues on hand hygiene practice among healthcare workers in the greater Kampala metropolitan area, Uganda: study protocol for a cluster randomized trial

**DOI:** 10.1186/s12913-021-06082-3

**Published:** 2021-01-26

**Authors:** Richard K. Mugambe, Jane Sembuche Mselle, Tonny Ssekamatte, Moses Ntanda, John Bosco Isunju, Solomon T. Wafula, Winnifred K. Kansiime, Prossy Isubikalu, David Ssemwanga, Habib Yakubu, Christine L. Moe

**Affiliations:** 1 Department of Disease Control and Environmental Health, School of Public Health, Kampala, College of Health Sciences, Makerere University, P.O. Box 7072, Kampala, Uganda; 2 Programs Department, WaterAid Uganda, P.O. Box 11759, Kampala, Uganda; 3 College of Computing and Information Sciences, Makerere University, P.O. Box 7062, Kampala, Uganda; 4 Department of Agricultural Extension and Innovations, School of Agricultural Sciences, College of Agriculture and Environmental Sciences, Makerere University, P.O. Box 7062, Kampala, Uganda; 5 Environmental Health Department, Ministry of Health, P.O Box 7272, Kampala, Uganda; 6 The Center for Global Safe Water, Sanitation and Hygiene, Rollins School of Public Health, Emory University, 1518 Clifton Rd. NE, Atlanta, GA 30322 USA

**Keywords:** Hand hygiene, Healthcare workers, Hand hygiene interventions, Behaviour centred design, Uganda

## Abstract

**Background:**

Hand hygiene (HH) among healthcare workers (HCWs) is critical for infection prevention and control (IPC) in healthcare facilities (HCFs). Nonetheless, it remains a challenge in HCFs, largely due to lack of high-impact and efficacious interventions. Environmental cues and mobile phone health messaging (mhealth) have the potential to improve HH compliance among HCWs, however, these remain under-studied. Our study will determine the impact of mhealth hygiene messages and environmental cues on HH practice among HCWs in the Greater Kampala Metropolitan Area (GKMA).

**Methods:**

The study is a cluster-randomized trial, which will be guided by the behaviour centred design model and theory for behaviour change. During the formative phase, we shall conduct 30 key informants’ interviews and 30 semi-structured interviews to explore the barriers and facilitators to HCWs’ HH practice. Besides, observations of HH facilities in 100 HCFs will be conducted. Findings from the formative phase will guide the intervention design during a stakeholders’ insight workshop.

The intervention will be implemented for a period of 4 months in 30 HCFs, with a sample of 450 HCWs who work in maternity and children’s wards. HCFs in the control arm will receive innovatively designed HH facilities and supplies. HCWs in the intervention arm, in addition to the HH facilities and supplies, will receive environmental cues and mhealth messages. The main outcome will be the proportion of utilized HH opportunities out of the 9000 HH opportunities to be observed. The secondary outcome will be *E. coli* concentration levels in 100mls of hand rinsates from HCWs, an indicator of recent fecal contamination and HH failure. We shall run multivariable logistic regression under the generalized estimating equations (GEE) framework to account for the dependence of HH on the intervention.

**Discussion:**

The study will provide critical findings on barriers and facilitators to HH practice among HCWs, and the impact of environmental cues and mhealth messages on HCWs’ HH practice.

**Trial registration:**

ISRCTN Registry with number ISRCTN98148144. The trial was registered on 23/11/2020.

**Supplementary Information:**

The online version contains supplementary material available at 10.1186/s12913-021-06082-3.

## Background

Adequate water, sanitation and hygiene (WASH) is critical in the provision of quality health care in healthcare facilities (HCFs). Improved WASH infrastructure and practices in HCFs can significantly reduce the risk of healthcare acquired infections (HCAIs). Moreover, it increases trust and uptake of healthcare service while increasing efficiency and improved staff morale. In addition, all major initiatives to improve global health largely depend on basic WASH services in HCFs [1]. Despite this, many HCFs in low- and middle-income countries (LMICs) lack basic WASH infrastructure and where it exists the coverage is low. The recent Joint Monitoring Programme (JMP) report on WASH in HCFs indicates that approximately 14% of HCFs had limited water services while 21 and 16% respectively had no sanitation and hygiene services, impacting nearly 2 billion people [2]. The lack of WASH infrastructure compromises the ability to provide safe and quality healthcare services, and places both healthcare workers (HCWs) and patients at a considerable and yet preventable health risk of HCAIs. Indeed there is a strong biological plausibility that pathogens responsible for HCAIs are more prevalent in facilities with substandard WASH services [3], and mothers and neonates are at the greatest risk of getting exposed to HCAIs [4–6].

Hand hygiene, a critical component of standard infection prevention and control (IPC), is a leading simple, crucial and inexpensive measure for preventing HCAIs [7–9]. However, there is limited evidence on efficacious interventions that have the potential to increase hand hygiene compliance in HCFs [10, 11]. Yet, hand hygiene compliance among HCWs in HCFs remains low especially in low and middle income settings [10, 11]. There is an avalanche of reasons for suboptimal hand hygiene practices and these defer by settings and resources available. For example lack of appropriate infrastructure and equipment [12]. Other factors for poor hand hygiene compliance include belonging to a certain professional category, working in certain departments, understaffing and wearing gloves and gowns [13]. The barriers are not any different in Uganda, where low compliance to hand hygiene among HCWs has been related to lack of knowledge of the transmission risk, and limited hand hygiene infrastructure and supplies [14, 15].

In Uganda, most of the existing research on WASH in HCFs has mainly focused on assessing WASH status [14–17] but there has been generally a lack of understanding of the barriers and facilitators to hand hygiene among HCWs, and research on simple, effective and inexpensive interventions to enhance hand-hygiene practices among HCWs is limited. In other settings, previous studies have shown that the use of mobile phone technology in health messaging (mhealth) has great potential to promote health care including improving HCWs’ adherence to standard guidelines [18, 19]. There is growing evidence that mhealth interventions are critical in improving patients’ adherence to treatment as well as adherence of HCWs to treatment guidelines. Despite the impact of mHealth in HCWs’ adherence, there is limited evidence about the use and effect of the mhealth in enhancing compliance to WASH practices including hand hygiene among HCWs [20, 21]. In addition, there is also evidence that nudges (environmental cues) in schools, have the potential to improve handwashing with soap among school-aged children [22], however, such evidence is limited for HCFs. Complex interventions such as use of HCF wide poster campaign, combined with performance feedback and alcohol-based hand rub (ABHR) placed at every bedside; introducing ABHR accompanied by education/training; applying social marketing strategies as well as using multiple strategies including involvement of staff in planning activities have been suggested as interventions for improving adherence to hand-hygiene guidelines among HCWs [23]. However, there is limited evidence on the effect of simple inexpensive mhealth and nudges/environmental cues related interventions on compliance to hand hygiene among HCWs.

### Study objectives

#### General objective

To determine the impact of mobile phone WASH text messages and environmental cues on hand hygiene practice among HCWs in the Greater Kampala Metropolitan Area (GKMA).

#### Specific objectives

1. To explore the facilitators of hand hygiene among HCWs in HCFs in the GKMA.
2. To explore the barriers to hand hygiene among HCWs in HCFs in the GKMA.
3. To determine the impact of mhealth messages and environmental cues on hand hygiene practices among HCWs in HCFs in the GKMA.

## Methods

### Study setting

The study will be conducted in the GKMA which includes Kampala, Wakiso and Mukono districts. According to the Uganda Bureau of Statistics (UBOS), Kampala has a day time population of approximately 4 million people and a night time population of approximately 1.5 million [24]. The rate of urbanisation in Kampala and the neighbouring districts of Wakiso and Mukono is so high, and these areas are experiencing many urban challenges including growth of slums, which are characterized by poverty, poor living conditions and limited access to various services [25, 26]. In partnership with the Ministry of Health (MOH), Ministry of Water and Environment (MWE), WaterAid Uganda and Kampala Capital City Authority (KCCA), we recently conducted a WASH assessment in 63 HCFs in the GKMA, which indicated significant gaps in WASH [16]. This proposed study will be building upon the evidence from that assessment, as well as priorities and needs that have been identified by stakeholders. The intervention will be premised on the understanding that mobile phone usage is very high among HCWs. Indeed, according to the Uganda National Information Technology Agency (NITA), 70.9% of adult Ugandans own a mobile phone, and ownership is even higher in professional groups of people including HCWs [27]. As of 2018, the number of Government and Private not for Profit (PNFP) HCFs in the 3 districts of the GKMA is as provided in Table 1 [28].

**Table 1:** Number of HCFs in Kampala, Wakiso and Mukono districts

District	Hospitals		HC IVs		HC IIIs		Total
	Government	NGO	Government	NGO	Government	NGO	
Kampala	5	9	4	3	8	12	41
Mukono	0	1	2	1	13	1	18
Wakiso	1	3	5	0	21	16	46
TOTAL	6	13	11	4	42	29	105

### Study design

In Uganda, the healthcare system is organised into a four-tier system with hospitals and health centres (HCs) of levels IV, III and II [29]. General hospitals (catchment population 500,000 people) provide preventive, promotive, curative, maternity, and inpatient health services and surgery, blood transfusion, laboratory, and medical imaging services. HC IVs have a target population of 100,000 people and are responsible for preventive, outpatient health services, maternity, inpatient health services, emergency surgery and blood transfusion, and laboratory services. HC IVs provide all the services of HC IIIs except emergency surgery. The study will be carried out in public HCs of level III and IV in Wakiso and Mukono districts, using a cluster-randomized trial (CRT) design. The study will be restricted to public HC IVs and IIIs because these offer affordable Maternal, New-born and Child Health (MNCH) services to the majority of the population in the GKMA [14], and only HCFs in Wakiso and Mukono will be studied since they are likely to have similar characteristics as they are more rural and receive less funding compared to those in Kampala. The intervention will be preceded by a formative study which will be conducted in the Kampala, Mukono and Wakiso districts. The reason for focusing on HCFs and departments that offer MNCH services is because mothers and neonates are at a greater risk of getting exposed to HCAIs [4–6], and there is therefore critical need to improve the status of WASH in maternal and children’s wards.

**Fig. 1:**
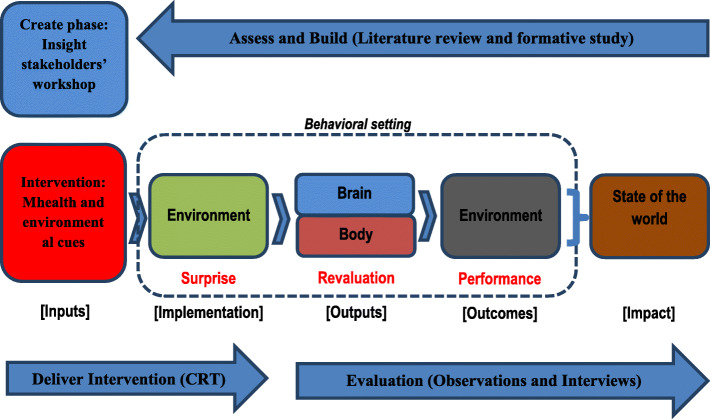
Study steps adapted from the Behaviour Centred Design model for behaviour change [30]

The study will be guided by the “Behaviour Centred Design (BCD)” model and theory for behaviour change [30], which guides hand hygiene program design through the “ABCDE” (Assess, Build, Create, Deliver and Evaluate) steps (Fig. 1). The model provides the BCD process on the outside and the theory of change on the inside. The theory suggests that, the right behavioral response depends on the behavior settings (physical, social and temporal context) in which individuals find themselves [31], and that three causal links must be made: from the environment (modified by an intervention) to psychological change in the target population (body and brain), to performance of the target behaviors (which results in changes to the state-of-the-world).

Prior to the implementation of the CRT, the investigators will obtain a list of HCFs and healthcare providers from the respective district health offices. This list will be used to randomly assign the clusters (HCFs) to the intervention and control groups. Once the clusters have been assigned, the study participants in the maternal and children’s wards within the selected HCFs will be informed about the purpose of the study and recruited in the study after the consenting process.

### Study approach

This study will be implemented following these stages;

#### Assess - document existing hand hygiene behaviour

In determining the barriers and opportunities for enhancing HCWs’ hand hygiene practices, this study component will involve collating and reviewing available published literature on hand hygiene provisions and drivers among HCWs in HCFs in Uganda and beyond. The literature review will be conducted with the help of a literature review guide. The findings from the literature review will guide the “build” (formative) research phase.

#### Build - formative phase

The formative research phase will address objectives 1 and 2 of the study, and the findings will inform the design and implementation of the intervention (mhealth and environmental cues on hand hygiene). The purpose of this formative phase is to understand the drivers of hand hygiene among HCWs. During this phase, we shall conduct key informants’ interviews (KIIs) and semi-structured interviews using developed guides. Multi-variation sampling will be conducted to select participants for the interviews and the sample sizes will be determined based on theoretical saturation in the data [32]. Structured observation checklists will be used to establish the status of HCF hygiene infrastructure and hand hygiene behaviour of HCWs.

#### Create - stakeholders’ workshops to develop and refine the intervention

A stakeholders’ workshop shall be conducted to share findings from the literature review, formative research and discuss the contextual mechanisms of designing and implementing the mhealth and environmental cues intervention. Stakeholders will give their ideas on how best the mhealth intervention should be designed and used to disseminate messages to HCWs. The choice of the environmental cues (colours or pictures to paint around hand hygiene stations, branding of mirrors etc) and the mhealth (mobile texting approach or use of social media such as Facebook or Whatsapp etc) for communicating behavioural change messages will depend on the guidance of the WASH stakeholders, subject area experts and reviewed literature. The stakeholders will include MWE, MOH, Kampala Capital City Authority (KCCA), Non-Government Organisation (NGOs), representatives from local authorities, District and HCFs’ IPCcommittees, WASH consultants as well as HCWs from different levels of HCFs in the GKMA.

During the stakeholders’ workshop, the World Café approach [33, 34] will be used, and 7–8 participants will sit around tables to internalize the findings from the formative research. Within the different groups, participants will use findings from the formative research to develop themes which will be used to develop insights, appealing stories that link the theme to the behaviour (hand hygiene among HCWs). The insights will be formulated to include: the behaviour to change; the character to influence; the motives to encourage the behaviour of that character as well as the direct or indirect reinforcement of social norms to encourage behaviour change. The insights from the different groups will be presented, and participants will choose the best 4–5 insights based on richness (how many of the formative research findings are captured by the insight), power (how strong the logic is in linking the insight to the target behaviour), plasticity (how likely the idea on which the insight is based could be changed by the intervention), novelty (is it a surprise and acceptable to the target population). Using “how might we questions”, participants will identify the campaign activities, tools and touch points that will enable HCWs to practice hand hygiene, change the settings, and create social norms.

The workshop will be used to develop and refine the intervention for implementation in the HCFs, using the workshop guide.

#### Delivery of the intervention

i. Intervention design

The study will be implemented through a CRT targeting HCWs working in maternity and children’s wards. The intervention will be implemented over a period of 4 months, through a partnership with WaterAid Uganda, MOH, KCCA, MWE as well as selected HCFs. The design of the study was guided by the checklist for standard protocol items recommended for international trials (SPIRIT), which is provided in Additional file 1.

ii. Trial interventions

In both the intervention and control HCFs, simple innovatively designed hand hygiene facilities (tapped plastic jerricans with a stand and basin) will be provided (where needed) in delivery rooms, post-natal wards, and children’s wards. Soap and ABHR will be provided at all hand hygiene stations. Additionally, ABHR will be provided at points of patient care and on medication trolleys. Hand hygiene demonstrations using the “glo germ” gel will be done in both study arms at the beginning of the study. The choice of the departments/wards for the interventions is based on the understanding that mothers and neonates face the greatest risk of infections related to poor WASH and IPC measures in HCFs [35], but these wards will also allow the research team to maximize the observations of hand hygiene opportunities while standing within a radius of about 5–10 m [36].

In the intervention HCFs, besides the provisions in the control group, two interventions, mhealth and environmental cues (nudges) that have been successful in enhancing adherence to treatment guidelines and enhancing hand-hygiene in school going children [19, 22] respectively will be used. The choice of the specific mhealth and nudges intervention, and the frequency of exposure to mhealth messages among the study participants will be determined in the insight workshop. The mhealth intervention messages will be designed with a focus on: benefits of hand hygiene; when to do hand hygiene; how to do hand hygiene and how to protect others. However, the messages will also have educational jokes and response prompts where HCWs with the highest number of responses will win hand hygiene supplies. This will be critical in enhancing participant retention and complete follow-up. The educational messages and jokes in the mhealth intervention will be guided by the results from the formative study.

The mhealth messages will be sent to study participants using either short message service (sms) or whataspp, depending on the decision of the stakeholders in the stakeholders’ workshop. In case stakeholders choose using the sms option, messages will be sent to participants using RapidSMS, which is a free and open-source framework for dynamic data collection, logistics coordination and communication. The service will enable a two-way communication between HCWs using basic SMS mobile phone technology. RapidSMS will be installed on a secure Linux server running Ubuntu 18.04.5 LTS with the following hardware specifications: (1) Intel Core i7-8700T 2.40 GHz processor with 16.0 GB of RAM, and (2) An MTCBA-GF4 modem attached via a serial interface. RapidSMS dependences that include Python and Django web framework will also be installed on the same machine. For compatibility, we shall install Python 3.7.3 and Django 2.2. For communication between the modem and RapidSMS, we shall install Kannel, which is a free and open source SMS gateway compatible with RapidSMS. With Kannel’s SMS Delivery Report functionality, we shall be able to track the status of messages sent to HCWs. We shall configure the system to automatically send mhealth messages to HCWs at specific weekly intervals. Our server will enable HCWs to respond to the messages sent by the system. SMS received by the system from HCWs will be toll free thus HCWs will not incur any costs when responding to mhealth messages. Responses from HCWs will be securely stored in MySQL database on the server; this will enable further analytics on the data. Authorized access to the data will be available via secure login into the system. The data will be available and downloadable in CSV format to enable further processing by third party statistical tools. The software can track message history and delivery, and this will be critical in understanding the proportion of HCWs receiving and responding to sms messages on a daily basis.

iii. Randomization and Trial arms

Using a list of level III and IV HCFs in the study districts [28], the project coordinator will use computer-generated random numbers to assign HCFs to the intervention and control arms. All HCWs from the same HCF will be allocated to the same group as the randomized HCF in which they are working. HCWs will be enrolled by research assistants (RAs) under the guidance of the principal investigator, and they will be masked off the intervention they are receiving.

iv. Masking/ blinding

The data analysts and the data collectors will be blinded to the group assignment. In order to achieve blinding for the data collection team, independent teams will collect data from either arm of the trial. In addition, data collectors will be trained separately.

xxii. Inclusion and exclusion criteria

The study will enrol participants working in the maternal and pediatric wards of selected HCFs, and Table 2 summarizes the inclusion and exclusion criteria.

vi. Intervention monitoring

**Table 2:** Inclusion and exclusion criteria

Inclusion criteria	Exclusion criteria
• Age of 18 and above • Both male and female • Minimum of six months experience at the HCF. • HCWs with appointment letters to work at the selected HCF • Full-time staff at the selected HCF • Informed written consent to participate in the study	• All HCWs in maternal and children’s wards of selected HCFs, who will be on leave at the time of the baseline • All HCWs workers in maternal and children’s wards of selected HCFs, who will be so sick at the time of the baseline • Refusal to give informed written consent to participate in the study.

For quality delivery of the intervention and ensuring that results are acceptable to the different stakeholders, an intervention support team will be in place to provide technical guidance and support. The intervention support team or data monitoring committee (DMC) will be comprised of 6 members, with a representative from the Environmental Health Department at the MOH, KCCA Public Health department, MWE, NGOs, HCFs and private sector. The independent DMC will be chaired by a representative from the Environmental Health division at the MOH.

### Outcome measurement

The primary outcome will be the proportion of utilized hand hygiene opportunities (number of critical times hand hygiene is observed to be done with soap and water or ABHR) out of the total number of observed hand hygiene opportunities. The secondary outcome will be *E. coli* concentration levels in hand rinsates from HCWs, which indicates recent fecal contamination and hand hygiene failure.

We shall determine the two outcome variables using two approaches. Firstly, observations based on the WHO’s 5 moments for hand hygiene which include: before touching a patient; immediately before performing an aseptic procedure; immediately after an exposure risk to body fluids (and after glove removal); after touching a patient and his or her immediate surroundings when leaving as well as after touching any object or furniture in the patient’s immediate surroundings, when leaving - even without touching the patient [37]. Hand hygiene observations will be conducted during the baseline, midline and end-line surveys. The hand hygiene observations will be done in the mornings (8 am – 12 pm) and evenings (4 pm – 8 pm) since these are considered to be peak treatment hours [36]. Within the targeted time, 20 hand hygiene opportunities will be observed for each of the selected HCWs (corresponding to 10 patients each with 2 hand hygiene opportunities, which will include before touching a patient and after touching a patient), and the used and missed opportunities will be recorded. Therefore, a total of 9000 hand hygiene opportunities will be observed by experienced researchers (HCWs or environmental health officers), who will observe from the least obtrusive point within a radius of 5–10 m of the patient wards [38].

Secondly, hand rinsates from HCWs participating in the CRT will be collected immediately after the hand hygiene observations during baseline and end-line. The samples (hand rinsates) will be collected by a team of trained environmental health officers. During sampling of hand rinsates, HCWs will put their hands, one at a time in Whirl-Pak bags containing distilled water. The HCWs will wash their hands by rubbing the inner hands for about 2 min and the enumerator massaging their hands from outside the whirl pak bag to remove any potential pathogens. Aseptic techniques will be used to collect duplicate samples from each of the study participants (HCWs) during baseline, midline and end-line. The samples will be stored in sterile plastic bottles and transported on ice to the laboratory for further analysis within 4 h. The hand rinsates will be analysed for *Escherichia coli (E.coli)*, to establish if there are differences in the levels of contamination on the hands of the HCWs before and after the intervention. During analysis, hand rinsates will be diluted using the ratios of 1:1, 1:10 and 1:100. The membrane filtration method (with Chromocult agar) will be used to concentrate samples, and incubation will be done at 37 °C for 24 h. Colonies of *E.coli* will be identified by their dark blue to violet colour, and they will be counted and recorded per 100 ml of sample.

### Data collection methods, respondents and sample size

i. Key informants’ interviews (KIs)

A total of 30 KIs selected based on their knowledge, position and experience on WASH aspects in HCFs will be conducted. KIs will include supervisors/managers of HC IIIs and IVs, nurses, administrators, Environmental Health Officers as well as officials from the MOH and district health office. KIs interviews will be used to explore the barriers and facilitators of hand hygiene in HCFs, and this will be done using a KIs’ interview guide, which is provided in Additional file 2.

ii. Semi-structured interviews

A total of 30 semi-structured interviews with HCF managers will be conducted, to assess barriers and facilitators to hand hygiene, motives of hand hygiene, social norms related to hand hygiene, behavioural settings and touch points. This will be done using a semi-structured interview guide provided in Additional file 3.

iii. Structured observations in HCFs

As part of the formative study, observations using a structured observation checklist (Additional file 4) will be conducted in 100 HCFs to assess the hand hygiene infrastructure, roles, presence of hand hygiene supplies, functionality of facilitates, and norms in maternity and children’s wards.

iv. Cluster randomized trial

A sample size of 30 clusters [HC IIIs and IVs] (15 HCFs under the intervention and 15 HCFs under the control arm) with 15 HCWs per HCF will allow us to detect a 20% increase in the proportion of HCWs that practice hand hygiene (hand washing/hand rub) at the 5 critical moments at 4 months between the control and the intervention groups. The calculation of the sample size is based on the following assumptions: a 25% and 5% increase in hand hygiene in intervention and control group respectively, a standard normal deviate of at 95% confidence level (1.96), a standard normal deviate at 80% power level (0.84), the proportion of HCWs practising hand hygiene in the control group (0.747) from an infection control study in HCFs in Arua district, Northern Uganda [39] and a design effect of 2.0 to account for the fact that there would be clustering of HCWs within HCFs. This gives a sample size of 426, however, in order to achieve a consistent cluster size, the sample size will be rounded off to 450 HCWs participants, with 225 in each of the study arms. The study will recruit 450 HCWs (doctors, clinical officers, nurses and midwives) who work in maternity and children’s wards. In each HCF, the study will target 13 nurses/midwives and 2 Doctors/Clinical Officers.

### Evaluation

We shall conduct process and outcome evaluations. The process evaluation will be conducted at the midline to understand how the intervention has been implemented. The process evaluation will seek to establish: whether the intervention is being implemented according to plan; whether the intervention is working or not; how many HCWs will be getting exposed to the touch points; how many HCWs will be responding to the mhealth intervention as well as the aspects of the intervention that participants will have liked. The baseline, midline and end-line evaluations will be conducted using a tool provided as Additional file 5.

**Fig. 2:**
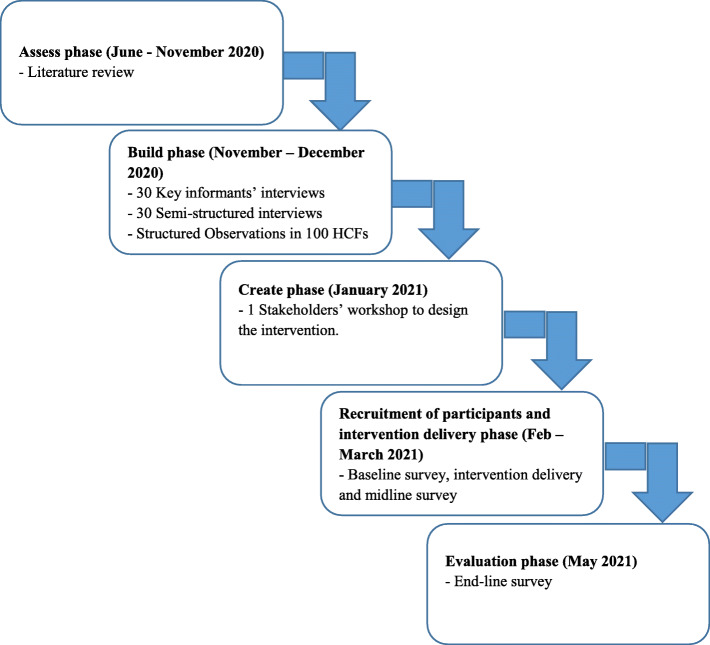
Project phases and timing

To assess the outcome of the intervention, a structured questionnaire will be administered at baseline, midline (2 months after starting intervention) and end-line (4 months) as indicated the participants’ timeline in Fig. 2. During surveys, social demographic, knowledge and attitudes questions will be collected from all participating HCWs through an interviewer administered questionnaire. The questionnaires will be administered to the HCWs before the hand hygiene observations. In order to maintain systematic consistency for measurement indicators, the same research tools used at baseline will be used for mid-term, end-line and impact evaluation. In addition, observations of hand hygiene practice among the HCWs will be done at baseline, midline and end-line using an observation tool. Hand rinsates will be collected and analysed, and the results will be entered in a hand rinsates form. All the data collection tools to be employed will be guided by the WHO’s 5 moments for hand hygiene [37].

### Data management and analysis plan

#### Data management

Data will be collected electronically using tablets or phones preloaded with Open Data Kit software. Data capture forms with in-built restrictions plus logical checks to minimize errors and missing data at collection will be designed by the study data management team. The data collected from the field will be submitted to the cloud server on a daily basis, and only accessible by the data management team for data security purposes. The data will be downloaded in a comma-separated value (csv) format, and then exported to STATA version 14 after which consistency checks programs will be run daily and error reports produced. The generated errors will be sent back to the field team for clarification and cleaning. The data manager will be responsible for the security of the data and will back it up on the cloud server on a daily basis to avoid its loss.

Study progress will be monitored by comparing numbers accrued against those expected. Numbers accrued will further be verified against the physical counts from the field team. Progress reports will be provided to the study team on a daily basis for purposes of monitoring the progress. There will be a data quality team which will monitor the data collection activity and carry out re-interviews on a randomly selected percentage of the records to further ensure quality data is collected. Data will be treated as confidential by the study personnel and all records will be kept secure in locked filling cabinets and offices will be treated with utmost confidentiality.

#### Data analysis

i. Qualitative analysis (formative stage)

The KIIs will be transcribed verbatim by two experienced transcribers. The typed transcripts will then be read several times by all members of the study team who will then develop codes. Code book definitions will be based on the objectives of the study while integrating in emerging themes from the data. The code book will be discussed and agreed upon by the study team. Then two experienced research assistants (RAs) will code the articles using ALTAS-ti software to ease further analysis. The code reports will then be read and discussed by all the investigators who will agree on both codes and categories. Then, codes will be grouped into categories, themes and subthemes [40].

ii. Quantitative analysis (effect of intervention)

The effect of the intervention will be evaluated at two time-points: midway the intervention, and immediately after the completion of the intervention period to assess the short-term effects on hand hygiene. Data from all surveys will be double entered into Open Data Kit (ODK) collect, and statistical analysis will be carried out using Stata version 14. We will calculate the prevalence of cluster level hand hygiene at baseline and follow-up for each HCF. Mixed sub-group analysis will be stratified by sex, cadre, experience, and level of training. We shall run multivariable logistic regression under the generalised estimating equations (GEE) framework to account for the dependence of hand hygiene on the intervention in a marginal way. This approach will give population average estimates for effect of intervention while accounting for other covariates in the model. An exchangeable correlation structure will be considered. Table 3 provides a summary of the study objectives, methods and data analysis procedures.

**Table 3:** Summary of study objectives, methods and data analysis procedures

Objectives	Study population	Data collection methods	Indicators	Data analysis
Explore the facilitators for enhancing healthcare workers’ hand hygiene practices in HCFs in the GKMA.	Healthcare workers in selected HCFs and district leaders.	Key informant interviews	Facilitators of Hand hygiene among HCWs	i. Thematic content analysis
		Semi-structured interviews	Facilitators of Hand hygiene among HCWs	i. Thematic content analysis ii. Descriptive statistics
	Healthcare facilities	Observations	Facilitators of Hand hygiene among HCWs	i. Descriptive statistics
Explore the barriers for enhancing healthcare workers’ hand hygiene practices in HCFs in the GKMA.	Healthcare workers in selected HCFs.	Key informant interviews	Barriers of Hand hygiene among HCWs	i. Thematic content analysis
		Semi-structured interviews	Barriers of Hand hygiene among HCWs	i. Thematic content analysis ii. Descriptive statistics
	Healthcare facilities	Observations	Barriers of Hand hygiene among HCWs	i. Descriptive statistics
Determine the impact of mobile phone WASH messages and environmental cues on hand hygiene practice among health workers in HCFs in the GKMA.	Healthcare workers in selected HCFs.	i. Observations of healthcare workers ii. Laboratory analysis of hand rinsates for *E.coli*	i. Percentage change (increase or decrease) in the used hand hygiene opportunities ii. Percentage change (increase or decrease) in the number of E.coli colonies/100 ml of samples of hand rinsates	i. Use of statistical tests - McNemar test - Paired t-test - GEE Logistic regression

### Quality assurance/ quality control of data and collection

Experienced RAs including observers will be recruited from a well-established network of RAs that have participated in successful research projects. These will be trained on research ethics, research design and how to minimize bias. Prior to data collection, there will be a pre-test of the data collection tools to ensure they are valid and reliable. This will also enable RAs to familiarise with the data collection tools and also correct any errors if discovered. Pretesting will be in Nyimbwa health centre IV, Luweero district. This HCF has been purposively selected because it shares similar characteristics with some of the HCFs in the GKMA. A quality control team will be instituted whose role will be to ensure that RAs adhere to the approved study procedures. To ensure quality in quantitative data entry, the data entry screen will be designed with skips and restrictions. In case of samples for hand rinsates, a duplicate sample of the hand rinsates will be obtained for validation of results.

### Dissemination plan

Study findings will be presented at appropriate international conferences and submitted for peer-reviewed publication. Besides, study findings will be disseminated in stakeholders’ meetings and to the HCWs and managers in study HCFs.

## Discussion

Hand hygiene is a paramount intervention in the prevention of healthcare acquired infections. However, the rate of adherence to hand hygiene during patient care remains as low as 15% among HCWs [41]. Besides, only a few studies have attempted to holistically understand the facilitators and barriers to hand hygiene among HCWs, and the impact of use of mobile phone text messages and environmental cues [21]. Therefore, our study will explore the facilitators and barriers to adherence to hand hygiene, and the impact of mhealth and environmental cues on hand hygiene practices among HCWs working in maternal and children’s wards.

Our study will be a cluster randomized trial in which HCWs in public HCFs in the GKMA will be assigned to the intervention and control arms. Cluster randomized trials have the potential to minimise potential biases such as confounding, and are therefore considered as the gold standard for generating the highest level of evidence [42]. HCWs in both the intervention and control arms will be exposed to simple innovatively designed hand washing facilities (tapped plastic jerricans with a stand and basin) which will be placed in delivery rooms, post-natal and children’s wards. Delivery rooms, post-natal and children’s will be considered since they present mothers, children and HCWs with an elevated risk to healthcare acquired infections, especially blood borne pathogens such as HIV, hepatitis B virus, *Staphylococcus aureus* and *Streptococcus pneumonia* [43, 44].

Though not widely studied, having simple hand washing facilities such as plastic jerrycans and tippy taps have been shown to increase adherence to hand hygiene at critical times at community level [45, 46]. Therefore, our study will utilise this intervention based on the notion that having simple innovatively designed hand hygiene facilities and the required supplies will increase the level of adherence. In addition to the interventions in the control arm, HCWs in the intervention arm will be exposed to environmental cues and mobile text messages on hand hygiene. Also, evidence from a simulation hospital study in a US medical centre and affiliated medical school indicated that environmental cues subconsciously influenced the hand hygiene practice [47]. Similarly, mobile text messages have also been reported to influence behaviour change in a number of healthcare interventions [48, 49]. However, their impact on promotion of hand hygiene in HCF settings is still understudied [21]. Mobile text messages are anticipated to promote adherence to hand hygiene by reminding HCWs to wash hands or use ABHR during the critical moments of patient care.

The impact of the environmental cues and mhealth intervention will be assessed based on adherence to the WHO’s 5 moments for hand hygiene [50] and *Escherichia coli* (E.coli) concentration levels in hand rinsates from HCWs. The WHO 5 moments of hand hygiene have widely been used in evaluating hand hygiene interventions in healthcare settings [50–52]. Similarly, *E. coli* has also been used as an indicator for measuring the effectiveness of hand hygiene in HCFs [53–55].

### Strengths and limitations of the study

To the best of our knowledge, this may be the first study to evaluate the impact of environmental cues and mhealth on hand hygiene practice among HCWs. However, observations of hand hygiene practice among HCWs are likely to be affected by social desirability bias. To solve this challenge, RAs that will undertake the observations will be trained, standard WHO observation tools will be used, and where possible two observers will be used to observe hand hygiene behaviour simultaneously. In addition, our findings are likely to be influenced by a change in hand washing behaviour, which could be attributed to the recent COVID-19 pandemic. The effect of COVID-19 will be factored in during data analysis through stratification of data.

### Trial status

The study has received ethical approval from the Makerere University Higher Degrees Research and Ethics, and was consequently registered by the Uganda National Council of Science and Technology. Administrative permission to conduct the study has also been obtained from the 3 study districts. Currently, we are conducting the formative study phase, which is being implemented through literature review, key informants’ interviews, semi-structured interviews and observations in HCFs. The intervention is set to begin in February 2021.

## Supplementary Information

**Additional file 1.**

**Additional file 2.**

**Additional file 3.**

**Additional file 4.**

**Additional file 5.**

**Additional file 6.**

## Data Availability

This protocol doesn’t include any data yet, and readers are requested to ask the authors for data once available.

## Abbreviations

ABHR: Alcohol Based Hand Rub
BCD: Behavior Centered Design
CRT: Cluster Randomized Trial
DMC: Data monitoring committee
GEE: Generalized estimating equations
GKMA: Greater Kampala Metropolitan area
HCAIs: Healthcare acquired infections
HCFs: Healthcare facilities
HCWs: Healthcare workers
IPC: Infection Prevention and Control
JMP: Joint Monitoring Programme
KCCA: Kampala Capital City Authority
LMICs: Low and middle income countries
MOH: Ministry of Health
MWE: Ministry of Water and Environment
NAS: National Academy of Sciences
NGO: Non-Government Organization
ODK: Open Data Kit
SMS: Short message service
UNICEF: United Nations Children’s Fund
WASH: Water, sanitation and hygiene
WHO: World Health Organization
