# Supplementary material for: Impact of mhealth messages and environmental cues on hand hygiene practice among healthcare workers in the greater Kampala metropolitan area, Uganda: study protocol for a cluster randomized trial
Source: BMC Health Serv Res. 2021 Jan 26;21:88. doi: 10.1186/s12913-021-06082-3 (PMC7835669; doi:10.1186/s12913-021-06082-3)
Supplement: Supplementary file 2 — Additional file 2. [file 12913_2021_6082_MOESM2_ESM.docx]

## **Key informant interview guide for health facility managers and district officials**

**Study tittle:** **Improving hand hygiene practice among healthcare workers through mhealth and environmental cues in Kampala Metropolitan Area.**

Date of interview: ______/ _______/ ____________

Name of health facility: _______________________________

Venue: ____________________

Language of interview: _______________________

Time interview started: ________________ Time interview ended: _________________

Key informant interview identifier: _____________________________________

**Note: The Key informant** **participant’s identifier** should be created as follows: Type the initials - “KII” – followed by the name of the health facility where the interview has taken place (first 3 digits), followed by the date of interview in the format ***yy/mm/dd*** and the participant’s number (3 digits) assigned cumulatively. The participant’s identifier should end with a code showing the cadre of the provider as explained below. For example, if the first respondent is interviewed on December 20^th^, 2015 at ABC health facility; this participant’s identifier should be in the form: **KII/ABC/15/12/20/001/MD, where MD= Medical Doctor. Please use: NO = Nursing officer and enrolled nurses; MW = Midwife or MD = Medical doctor; LAB=Laboratory staff; CO=Clinical officer at the end of each participant’s number to designate the type of participant interviewed.**

***Interviewer:* Please obtain any additional details about the participant, e.g. his/her name and telephone contacts should be kept separately for any follow-up interviews that may be deemed necessary after the initial contact.**

| **Guiding questions** |
| --- |

**SECTION A: General questions**

1. What services are offered by this healthcare facility/ healthcare facilities in this district?

How do you ensure that quality healthcare services are provided in this facility?

- Probe for: safety of both the providers and clients, patient and provider satisfaction with available services and environmental hygiene

1. Is infection prevention and control a priority in this healthcare facility? How is it a priority? If not a priority, why not?

- What infection and prevention control precautions are prioritised in this healthcare facility? In this district? How are these prioritised? Probe for: availability of IPC supplies such as hand washing facilities, budget for IPC interventions, trainings, policies and guidelines
- Are WASH services part of quality of care for you? Are you satisfied with those services?

**SECTION B: Compliance to hand hygiene in the healthcare facility**

1. How important do you think is hand hygiene in a healthcare setting? Why/why not?

- What is usual practice of hand hygiene among healthcare workers in this health care facility?
- Are you concerned about spread of infections in healthcare facilities? Why? Which infections?

1. How important do you think is hand hygiene is for patients? Why/why not? Do you think healthcare providers in this healthcare facility prioritise hand hygiene?
2. How do you ensure compliance to hand hygiene among healthcare providers in this healthcare facility/ district?
3. There are healthcare facilities where providers comply with hand hygiene during all the critical moments while there are those where compliance to hand hygiene is poor.

- How would you rate this healthcare facility in terms of compliance with hand hygiene? **Probe:** Do you think hand hygiene in this healthcare facility/ healthcare facilities in this district is sufficient? If yes, why and if NO, why not? If not, what do you think most needs to be improved?
- Do you think all healthcare providers wash their hands during all the critical moments? If yes, what motivates them? If No, what challenges hinder them from practicing hand hygiene during all the critical moments?

1. What factors facilitate healthcare workers to practice hand hygiene (HH)? (Probe for *ways on how physical environment, and social environment in HCFs influences HH practice*, Brains related factors ( a - *knowledge on HH need, benefits and steps, b - motivation to practice HH due to fear, disgust, attract, affiliate, status etc, c - behaviour being habitual/social norm*), body related factors (Probe for a - *influence of HCWs’ socio-demographic characteristics on hand hygiene*, b - *influence of senses (smell and sight etc) on HH*, and c - *HCWs’ skills the facilitate HH*), behaviour settings related factors (probe for *a - factors related to the stage where HH takes place* b - *whether HCWs individual roles, identity or responsibilities influence HH practice,* c - *whether HCWs daily routine of activities influences HH practice*, d - *whether HH practice depends on presence of some objects and infrastructure, and if yes how*, e - *mechanisms through which HCWs receive messages related to HH practice*, f - *existing hand hygiene programs, and relevant hand hygiene/IPC policies).*

**Expectations on Hand hygiene in healthcare facilities**

1. Do you have expectations about hand hygiene in this healthcare facility/ies? If so, what are they?

- Probes: Do you think that your expectations are similar to those of the healthcare facility staff?

1. Do you feel supported by healthcare staff for getting access to those services?

- Are the WASH services similarly accessible to anyone in the facility?
- Do you think that healthcare staff and patients should have similar goals for the provision of WASH services? Is it the case?
- Do you think that healthcare workers follow the rules in place relative to hygiene standards? If yes, why and if No, why not?

1. How is the culture of hand washing in this healthcare facility? How do healthcare providers in this healthcare facility perceive hand washing?

**Policy guidelines and standards relating to IPC**

1. What are national policies, guidelines, and/or regulations surrounding healthcare facility infection control and hand hygiene? Are there specific guidelines hand hygiene?

- What are the regional/local guidelines for healthcare facility infection control and hand hygiene?

1. Does this healthcare facility have specific guidelines on hand hygiene? What do the guidelines stipulate? Are those policies/ guidelines enforced? If yes, how and what are the repercussions for not complying with the guidelines?

**Supply chain of IPC materials**

1. What is your healthcare facility’s source of IPC materials? Who maintains the supply?
2. Who is in charge of buying hand hygiene supplies?
3. What hand hygiene supplies are used currently in the healthcare facility? Availability? For health workers? For patients and caregivers?
4. Do you have a sufficient budget to get a continuous supply of hand hygiene supplies?

Are your suppliers reliable?

**Capacity building (hygiene education, workers training, leadership building)**

1. Do staff receive any training or education about hand hygiene? How often? Who is the trainer? What is the information source?
2. Do staff ever receive training in management, supervision, or budgeting?
3. Do patients and caretakers receive any training or education about hand hygiene?
4. Did you see any change in IPC services after those trainings? What changes?
5. Do you feel confident in your capacities to maintain adherence to hand hygiene in this health care facility? What makes you confident/unconfident?
6. What is your experience training or arranging training for others? What is your own experience receiving training related to WASH services or management?

**Recommendations**

1. What are your suggestions for improving hand hygiene in this healthcare facility? What interventions do you think would be best suited for this healthcare facility?
