# Supplementary material for: Impact of mhealth messages and environmental cues on hand hygiene practice among healthcare workers in the greater Kampala metropolitan area, Uganda: study protocol for a cluster randomized trial
Source: BMC Health Serv Res. 2021 Jan 26;21:88. doi: 10.1186/s12913-021-06082-3 (PMC7835669; doi:10.1186/s12913-021-06082-3)
Supplement: Supplementary file 3 — Additional file 3. [file 12913_2021_6082_MOESM3_ESM.docx]

## **Semi-structured interview guide for the formative study**

**Study tittle:** **Improving hand hygiene practice among healthcare workers through mhealth and environmental cues in Kampala Metropolitan Area.**

Date of interview: ______/ _______/ ____________

Name of health facility: _______________________________

Venue: ____________________

Language of interview: _______________________

Time interview started: ________________ Time interview ended: _________________

Interview identifier: _____________________________________

**Introductory questions**

1. What is hand hygiene? Have often do you practice hand hygiene?
2. What motivates you to practice hand hygiene?
3. Does the leadership of this HCF facilitate you to practice hand hygiene? If yes, how do they help you to practice hand hygiene?
4. Is the hand hygiene infrastructure in the HCF sufficient and functional? What factors affect the reliability and functionality of hand hygiene infrastructure? How does it support you in practicing hand hygiene?
5. What bars/prevents you from practicing hand hygiene in this HCF? What do you think is your major barrier?
6. How can hand hygiene be promoted? Probe for suggestions on what can be done to motivate HCWs, change behavioural settings, create social norms? Probe for names and positions of people who can be used as Trusted voices to promote hand hygiene.
7. Does your HCF have an infection prevention and control committee in place? Probe for the roles and activities of the IPC committee? Probe for frequency of the IPC activities and commitment of HCWs to the activities.
8. Do you have partners working with this HCF to improve WASH and IPC? Probe for the names of the partners and their roles?

**SECTION B: Compliance to hand hygiene in the healthcare facility**

1. How important do you think is hand hygiene in a healthcare setting? Why/why not?

- What is usual practice of hand hygiene among healthcare workers in this health care facility?
- Are you concerned about spread of infections in healthcare facilities? Why? Which infections?

1. How important do you think is hand hygiene is for patients? Why/why not? Do you think healthcare providers in this healthcare facility prioritise hand hygiene?
2. How do you ensure compliance to hand hygiene among healthcare providers in this healthcare facility/ district?
3. There are healthcare facilities where providers comply with hand hygiene during all the critical moments while there are those where compliance to hand hygiene is poor.

- How would you rate this healthcare facility in terms of compliance with hand hygiene? **Probe:** Do you think hand hygiene in this healthcare facility/ healthcare facilities in this district is sufficient? If yes, why and if NO, why not? If not, what do you think most needs to be improved?
- Do you think all healthcare providers wash their hands during all the critical moments? If yes, what motivates them? If No, what challenges hinder them from practicing hand hygiene during all the critical moments?

1. What factors facilitate healthcare you to practice hand hygiene (HH)?

(Probe for ways on how physical environment, and social environment in HCFs influences HH practice, Brains related factors ( a - knowledge on HH need, benefits and steps, b - motivation to practice HH due to fear, disgust, attract, affiliate, status etc, c - behaviour being habitual/social norm), body related factors (Probe for a - influence of HCWs’ socio-demographic characteristics on hand hygiene, b - influence of senses (smell and sight etc) on HH, and c - HCWs’ skills the facilitate HH), behaviour settings related factors (probe for a - factors related to the stage where HH takes place b - whether HCWs individual roles, identity or responsibilities influence HH practice, c - whether HCWs daily routine of activities influences HH practice, d - whether HH practice depends on presence of some objects and infrastructure, and if yes how, e - mechanisms through which HCWs receive messages related to HH practice, f - existing hand hygiene programs, and relevant hand hygiene/IPC policies).

**Expectations on Hand hygiene in healthcare facilities**

1. Do you have expectations about hand hygiene in this healthcare facility/ies? If so, what are they?

- Probes: Do you think that your expectations are similar to those of the healthcare facility staff?

1. Do you feel supported by healthcare staff for getting access to those services?

- Are the WASH services similarly accessible to anyone in the facility?
- Do you think that healthcare staff and patients should have similar goals for the provision of WASH services? Is it the case?
- Do you think that healthcare workers follow the rules in place relative to hygiene standards? If yes, why and if No, why not?

1. How is the culture of hand washing in this healthcare facility? How do healthcare providers in this healthcare facility perceive hand washing?

**Policy guidelines and standards relating to IPC**

1. What are national policies, guidelines, and/or regulations surrounding healthcare facility infection control and hand hygiene? Are there specific guidelines hand hygiene?

- What are the regional/local guidelines for healthcare facility infection control and hand hygiene?

1. Does this healthcare facility have specific guidelines on hand hygiene? What do the guidelines stipulate? Are those policies/ guidelines enforced? If yes, how and what are the repercussions for not complying with the guidelines?

**Supply chain of IPC materials**

1. What is your healthcare facility’s source of IPC materials? Who maintains the supply?
2. Who is in charge of buying hand hygiene supplies?
3. What hand hygiene supplies are used currently in the healthcare facility? Availability? For health workers? For patients and caregivers?
4. Do you have a sufficient budget to get a continuous supply of hand hygiene supplies?

Are your suppliers reliable?

**Capacity building (hygiene education, workers training, leadership building)**

1. Do staff receive any training or education about hand hygiene? How often? Who is the trainer? What is the information source?
2. Do staff ever receive training in management, supervision, or budgeting?
3. Do patients and caretakers receive any training or education about hand hygiene?
4. Did you see any change in IPC services after those trainings? What changes?
5. Do you feel confident in your capacities to maintain adherence to hand hygiene in this health care facility? What makes you confident/unconfident?
6. What is your experience training or arranging training for others? What is your own experience receiving training related to WASH services or management?

**Recommendations**

1. What are your suggestions for improving hand hygiene in this healthcare facility? What interventions do you think would be best suited for this healthcare facility?
