# Supplementary material for: Impact of mhealth messages and environmental cues on hand hygiene practice among healthcare workers in the greater Kampala metropolitan area, Uganda: study protocol for a cluster randomized trial
Source: BMC Health Serv Res. 2021 Jan 26;21:88. doi: 10.1186/s12913-021-06082-3 (PMC7835669; doi:10.1186/s12913-021-06082-3)
Supplement: Supplementary file 4 — Additional file 4. [file 12913_2021_6082_MOESM4_ESM.docx]

# **MWater Survey Tool.**

| **No.** | **Question** | **Response** | **Skip** |
| --- | --- | --- | --- |
| **HEALTHCARE FACILITY CHARACTERISTICS** | | | |
| 1. | **District** | 1. Kampala 2. Mukono 3. Wakiso |  |
| 2. | **Name of the healthcare facility** |  |  |
| 3. | **Level of the health facility** | 1. Hospital 2. HCIV 3. Health centre III |  |
| 4. | Ownership of the healthcare facility | 1. Private Not for profit 2. Public |  |
| 5. | **Location of HCF** | 1. Urban 2. Rural |  |
| 6. | GPS Location of HCF |  |  |
| **INFECTION PREVENTION AND CONTROL WASH PROGRAMMING** | | | |
| 1. | Does health facility have an IPC-WASH focal point person? | 1. Yes 2. No |  |
| 2. | If yes, is the IPC-WASH focal point person trained? | 1. Yes 2. No |  |
| 3. | Do you have an IPC-WASH programme with a clear annual activity plan? | 1. Yes 2. No |  |
| 4. | Does the health facility have an IPC-WASH committee? | 1. Yes 2. No |  |
| 5. | What is the composition of IPC-WASH committee? (Tick all that apply) | 1. Environmental Health Worker 2. Nurses/Midwives 3. Hospital Administrator 4. Lab staff 5. Clinical Officers or Doctors 6. Other (please specify) |  |
| 6. | Has any of the clinical officers who is a member of IPC-WASH committee been trained in IPC-WASH? | 1. Yes 2. No |  |
| 7. | Is the IPC committee functional (ask for minutes of Meetings)? If possible, take proof of IPC committee meetings (e.g., pictures of records) | 1. Yes 2. No |  |
| 9 | Is the IPC WASH committee supported by budget line? (Confirm) | 1. Yes 2. No |  |
| 10. | What is the current year budget line? |  |  |
| 11. | Does health facility have IPC-WASH Information Education and Communication materials on display? | 1. Yes 2. No |  |
| 12. | Please take pictures with examples of the IPC- WASH communication education materials |  |  |
| 13. | Do the health care workers frequently receive training regarding IPC in your facility? | 1. Yes 2. No |  |
| 14. | What is the frequency of these trainings? | 1. Monthly 2. Every quarter 3. Every 6 months 4. Yearly 5. Ad-hoc (not regular) 6. Other (please specify |  |
| 15. | How many staff have been trained in IPC-WASH within the last one (1) year? |  |  |
| 16. | Have administrators / managerial staff been trained regarding IPC- WASH in your facility? | 1. Yes 2. No |  |
| 17. | Does health facility give daily IPC-WASH talks to patients or family members to minimize the spread of COVID-19? | 1. Yes 2. No |  |
| 18. | Are the wards or waiting rooms well-lit and ventilated? | 1. Yes 2. No |  |
| 19. | Are staff who have received training on prevention and management of COVID-19 in this facility? | 1. Yes 2. No |  |
| 20. | If yes, which type of training has been received by the staff? Check all that apply | 1. Detecting COVID-19 (signs and symptoms) 2. Treatment of COVID-19 3. Testing of COVID-19 (Lab test) 4. Referral pathways of COVID-19 5. Others specify |  |
| **WATER SUPPLY** | | | |
| **Source and access** | | | |
| 1. | **Is the main source of water improved?** *Hint: IMPROVED WATER SOURCE IS: piped supply inside the building; piped supply outside the building; Tube well/ borehole; protected dug well; protected spring; Rain water* | 1. Yes 2. No |  |
| 2. | **What is the main water supply for the facility?**  *Hint: This refers to the water supply for general purposes, including drinking, washing and cleaning. In case of water being available from multiple sources, record the main source used in the outpatient area.* | 1. Piped supply inside the building 2. Piped supply outside the building 3. Tube well 4. Borehole 5. Protected dug well 6. Unprotected dug well 7. Protected spring 8. Unprotected spring 9. Rainwater collection 10. Tanker-truck 11. Surface water - river, dam, lake, pond, stream, canal, irrigation channels 12. Don’t know 13. Other (please specify) |  |
| 3. | **Where is the main water supply for the facility located?**  *Hint: On premises means within the facility grounds. This question refers to the location from where the water is accessed for use in the health facility.* | 1. On premises 2. Within 500 meters 3. 500 meters or further |  |
| 4. | Is water available from the main supply at the time of the survey? | 1. Yes, observed 2. Yes, reported but not observed 3. No |  |
| 5. | Is water available to staff? | 1. All the time 2. Only at times 3. Never |  |
| **Quantity** | | | |
| 6. | Is water available from the main source at the time of the survey? | 1. Yes 2. No |  |
| 7. | Are there times when the main water source is unavailable? | 1. Yes 2. No |  |
| 8. | Is water storage is sufficient to meet the needs of the facility for 2 days? | 1. Yes 2. More than 75% of needs met 3. Less than 75% of needs met |  |
| 9. | How much total water storage (in Liters) is available for use by the facility? (if known) |  |  |
| 10. | Is there routinely a time of year when the healthcare facility has severe shortage or lack of water? | 1. Yes 2. No |  |
| 11. | Which month of the year there are issues with water shortage generally? | 1. January 2. February 3. March 4. April 5. May 6. June 7. July 8. August 9. September 10. October 11. November 12. December |  |
| **Quality** | | | |
| 12. | Is water from main source regularly tested for microbial quality E.g. E-coli? | 1. Yes 2. No |  |
| 13. | If yes*,* when was the latest test done? |  |  |
| 14. | What is the latest microbiological test value? |  |  |
| 15. | If available, please take pictures of the latest records of water quality testing results |  |  |
| 16. | Is water currently being treated? | 1. Yes, regularly 2. Yes, occasionally 3. No |  |
| 17. | What is the current water treatment method being used? | 1. Chlorination 2. Filtration 3. Boiling 4. UV 5. Distillation 6. Purchase 7. Other (please specify) 8. No treatment 9. Don’t know |  |
| **SANITATION FACILITIES** | | | |
| **Access** | | | |
| 1. | Does the HCF have sanitary facilities? | 1. Yes 2. No |  |
| 2. | If yes, what is the main type of latrine/sanitary facility present in the HCF? | 1. Flush / Pour-flush toilet to sewer connection 2. Flush / Pour-flush toilet to tank or pit 3. Pit latrine with slab 4. Composting toilet 5. Flush / Pour-flush toilet to open drain 6. Pit latrine without slab/open pit 7. Bucket 8. Hanging toilet/latrine 9. No toilet /latrine 10. Other (please specify |  |
| 3. | **Are there separate toilets or improved latrines stances for men and women (patients/ caregiver) on the premises?**  *Hint: IMPROVED SANITATION FACILITIES include: flush/pour flush connected to piped sewer system; flush/pour flush connected to septic tank or pit latrine; ventilated improved pit latrines; pit latrines with slabs; composting toilets with slab.* | 1. Yes 2. No |  |
| 4. | **Is there at least one usable improved toilet designated for women and girls, which provides facilities to manage menstrual hygiene needs?**  *Hint: A toilet can be considered to meet the needs of menstrual hygiene management if it satisfies at least one of the following conditions: has a bin with a lid on it within the cubicle; has water available in a private space for washing.*  This refers to either staff or patient toilets. A toilet can be considered to meet the needs of menstrual hygiene management if it meets at least one of the following conditions: 1. a bin with a lid on it within the cubicle 2. water and soap available in a private space for washing | 1. Yes 2. No, female-only toilets do not have menstrual hygiene facilities 3. No, there are no female-only toilets |  |
| 5. | Is there at least one separate toilet or improved latrine stances for staff on the premises? | 1. Yes 2. No |  |
| 6 | **Is there at least toilet or improved latrine stance that meets the needs of people with reduced mobility?**  *Hint: A toilet or latrine can be considered accessible for people with limited mobility if it meets the following conditions: • can be accessed without stairs or steps; handrails for support are attached either to the floor or sidewalls; the door is at least 80 cm wide; and the door handle and seat are within reach of people using wheelchairs or crutches/sticks* | 1. Yes 2. No |  |
| 7 | Does the HCF have sanitation facilities clearly separated for staff, and patients/visitors and for male and female? | 1. Yes 2. No |  |
| **Quantity** | | | |
| 8. | On an average day, how many inpatients are at the healthcare facility? |  |  |
| 9. | On average, how many outpatients are seen per day? |  |  |
| 10. | How many toilets or improved latrine stances are available in the facility? |  |  |
| 11. | **How many usable toilets or improved latrine stances are available to inpatients?** *Hint: USABLE TOILET should be available, functional and private at the time of the survey or questionnaire. Toilets are available when within premises, doors are unlocked or with a key available at all times. To be functional, the hole or pit is not blocked, water is available for flush/pour flush toilets, and there are no cracks or leaks in the toilet structure. To be considered private, the toilet stall has doors that can be locked from the inside and there are no large gaps or holes in the structure. If any of these criteria are not met, the toilet/latrine is not counted as usable.* |  |  |
| 12 | **How many usable toilets or improved latrine stances are available to outpatients?** *Hint: USABLE TOILET should be available, functional and private at the time of the survey or questionnaire. Toilets are available when within premises, doors are unlocked or with a key available at all times. To be functional, the hole or pit is not blocked, water is available for flush/pour flush toilets, and there are no cracks or leaks in the toilet structure. To be considered private, the toilet stall has doors that can be locked from the inside and there are no large gaps or holes in the structure. If any of these criteria are not met, the toilet/latrine is not counted as usable.* |  |  |
| 13. | **Does the HCF have the required number of usable latrines for inpatients? Based on the numbers you just provided you have reported [expression] of latrines stances per inpatient. The maximum limit should be 20.**  *Hint: Adequacy of toilets or latrine stance in inpatient settings should be at least one toilet per 20 inpatients. If these conditions are met (based on the numbers of inpatients mentioned) then the indicator meets the target.* | 1. Yes, is less than 20 users per latrine stance 2. No, there are more than 20 users per latrine stance |  |
| 14. | **Does the HCF have the required number of usable latrines for out patients? Based on the numbers you just provided you have reported [expression] of latrines stances per Impatience. The maximum limit should be 40.** *Hint: Adequacy of toilets or latrine stance in outpatient settings should be at least one toilet per 40 outpatients. If these conditions are met (based on the numbers of outpatients mentioned) then the indicator meets the target.* | 1. Yes, is less than 40 users per latrine stance 2. No, there are more than 40 users per latrine stance |  |
| **Infrastructure** | | | |
| 15. | **Is faecal waste from toilets safely managed?** *Hint: If faecal waste is disposed in sewers, septic tanks or in covered pits, and no faecal matter is seen discharging out* | 1. Yes 2. No |  |
| **HYGIENE** | | | |
| **Hand hygiene facilities** | | | |
| 1. | Does the HCF have a functional hand washing facility within 5 metres of existing toilet blocks? | 1. Yes, with both water and soap 2. Yes, but only with water 3. Yes, but only with soap 4. No |  |
| 2. | **Does the Health Care Facility have a functional hand washing facility with soap and water or hand sanitizer at all points of care in the facility?** *Hint: WHO defines point of care as any location in the outpatient setting where care or treatment is delivered (e.g. Consultation rooms, Examination rooms, Triage rooms, Treatment rooms).• Othername…* | 1. Yes, with both water and soap/ hand sanitiser 2. Yes, but with only water 3. Yes, but with soap/hand sanitiser 4. No |  |
| 3 | Does the HCFs undertake routine hand hygiene compliance monitoring? | 1. Yes 2. No |  |
| 4. | If yes, how frequently is direct observation of hand hygiene compliance for health workers performed (or similar technique)? | 1. Never 2. Irregularly 3. Annually 4. Every six (6) months 5. Every 3 months or more often |  |
| 5. | Does the Health Care Facility have a functional hand washing facility with soap and water or hand sanitizer at the main entrance/gate? | 1. Yes, with both water and soap/ hand sanitizer 2. Yes, but with only water 3. Yes, but with soap/hand sanitiser only 4. No |  |
| 6. | If yes, is hand washing with soap and clean water or hand rub with a hand sanitizer enforced at the main entrance/gate prior to client's access to facility? | 1. Yes 2. No |  |
| **ENVIRONMENTAL CLEANLINESS** | | | |
| **Training and protocols** | | | |
| 1. | Is there a guideline or procedure (SOP) for cleaning surfaces and worktops in the service areas? | 1. Yes 2. No |  |
| 2. | Is there a visible cleaning roster or schedule specifying responsibility for cleaning tasks and frequency at which they should be performed? | 1. Yes 2. No |  |
| 3. | Have staff with cleaning responsibilities received training on WASH/IPC? | 1. Yes 2. No |  |
| **Equipment and supplies** | | | |
| 4. | Are there enough supplies for cleaning (detergents, mops, buckets, wheelbarrow, squeezers etc) in the wards and outpatients? | 1. Yes 2. No |  |
| 5. | Are there enough supplies used for cleaning in the Labour and Delivery ward? | 1. Yes 2. No |  |
| 6. | Is PPE available at all times and in sufficient quantity for all uses for all health care workers and cleaners? | 1. Yes 2. No |  |
| **Facility hygiene** | | | |
| 7 | Are the ward visibly clean and free from dust and soil? | 1. Yes 2. No |  |
| 8 | Are there uncleaned spills from bodily fluids (blood, urine, faeces, vomit, etc.) at points of care? | 1. Yes 2. No |  |
| 9 | Are beds, mattresses, pillows and/or mats cleaned between patients? | 1. Yes 2. No |  |
| 10 | Is open defecation practiced at this healthcare facility? Check and observe for indicators of open defecation | 1. Yes 2. No |  |
| 11 | Is there un contained solid waste on facility premises? | 1. Yes 2. No |  |
| **WASTE MANAGEMENT** | | | |
| **Segregation and storage** | | | |
| 12 | Is waste safely segregated into at least three labelled bins, including sharps waste, infectious waste and non-infectious general waste? *Hint: Safe segregation of waste: The bins should be colour-coded and/or clearly labelled, no more than three quarters (75%) full, and each bin should not contain waste other than that corresponding to its label. Bins should be appropriate to the type of waste they are to contain; sharps containers should be puncture-proof and others should be leak-proof (lined). Bins for sharps waste and infectious waste should have lids.* | 1. Yes 2. No |  |
| 13. | Is all infectious or sharps waste safely stored in a protected area before treatment or disposal? *Hint: Safely stored in a protected area: Fenced area protected from flooding; lined and covered pit > 30 m from water source; and no unprotected health care waste is observed. If waste removed off site, both the site and the holding area (minus the pit) should comply with the above requirements.* | 1. Yes 2. No |  |
| **Treatment and disposal** | | | |
| 14 | Is there arrangement by the health facility to safely treat/dispose of infectious waste? *Hint: Safe treatment of infectious or sharps waste: Methods considered to meet the basic service level include auto claving; incineration; burial in a lined, protected pit; and collection for medical waste disposal off-site.* | 1. Yes 2. No |  |
| 15 | Is there arrangement by the health facility to treat/dispose of sharp waste? | 1. Yes 2. No |  |
| 16 | Does the HCF have a functional incinerator for the treatment of infectious wastes and sharps? | 1. Yes 2. No |  |
| 17 | Does the health facility have a placenta pit? | 1. Yes 2. No |  |

**Assessment of Sinks and hand hygiene supplies**

**Please now walk to each room or area where patient care/treatment takes place in this ward (i.e. the point of care*) and complete the table below.**

|  | Room N°/ID | Total N° of beds in this room/area | N° of beds with handrub within arm’s reach | N° of sinks in this room/area (Functional. out of non-Funct) | N° of Func. sinks with clean water | N° of func. sinks with soap | N° of func. sinks with disposable towel | N° of func. sinks with clean water, soap, disposable towel | Total N° of handrub dispensers in this room/area | N° of fully-functioning and filled dispensers | N° of health-care workers encountered | N° of health-care workers encountered with handrub bottle in their pocket |  |
| --- | --- | --- | --- | --- | --- | --- | --- | --- | --- | --- | --- | --- | --- |
| A) All Patient rooms (Maternity ward, Children and Men’s wards) | | | | | | | | | | | | | |
| 1 |  |  |  |  |  |  |  |  |  |  |  |  |  |
| 2 |  |  |  |  |  |  |  |  |  |  |  |  |  |
| 3 |  |  |  |  |  |  |  |  |  |  |  |  |  |
| 4 |  |  |  |  |  |  |  |  |  |  |  |  |  |
| 5 |  |  |  |  |  |  |  |  |  |  |  |  |  |
| 6 |  |  |  |  |  |  |  |  |  |  |  |  |  |
| 7 |  |  |  |  |  |  |  |  |  |  |  |  |  |
| 8 |  |  |  |  |  |  |  |  |  |  |  |  |  |
| 9 |  |  |  |  |  |  |  |  |  |  |  |  |  |
| 10 |  |  |  |  |  |  |  |  |  |  |  |  |  |
| TOT | / |  |  |  |  |  |  |  |  |  |  |  |  |
| B) Treatment/Consultation rooms (ambulatory, day hospital, etc.) | | | | | | | | | | | | | |
| 1 |  |  |  |  |  |  |  |  |  |  |  |  |  |
| 2 |  |  |  |  |  |  |  |  |  |  |  |  |  |
| 3 |  |  |  |  |  |  |  |  |  |  |  |  |  |
| TOT | / |  |  |  |  |  |  |  |  |  |  |  |  |
| C) All toilets, food preparation areas, corridors and other areas with points of care* | | | | | | | | | | | | | |
| 1 |  |  |  |  |  |  |  |  |  |  |  |  |  |
| 2 |  |  |  |  |  |  |  |  |  |  |  |  |  |
| 3 |  |  |  |  |  |  |  |  |  |  |  |  |  |
| TOT | / |  |  |  |  |  |  |  |  |  |  |  |  |
| TOT |  |  |  |  |  |  |  |  |  |  |  |  |  |

TOT = total; N° = number

*Point of care: the place where three elements occur together: the patient, the health-care worker, and care or treatment involving contact with the patient and his surroundings.

**SECTION II: This section requires that you move around the entire facility.**

| S/n | **FACILITY OBSERVATION ITEMS** | **Response** |
| --- | --- | --- |
|  | Are there reminders and/or job aids posted that promote hand hygiene at this facility? | 1. Yes 2. No If No, go to 202 |
|  | If yes, describe what you saw |  |
|  | Do all healthcare providers wash their hands before examining patients? | 1. Yes 2. No 3. Not applicable (No clinical work in progress) |
|  | Do all healthcare providers wash their hands after examining patients? | 1. Yes 2. No 3. Not applicable (No clinical work in progress) |
|  | Do all healthcare providers wash their hands after touching the patient surroundings? | 1. Yes 2. No 3. Not applicable (No clinical work in progress) |
|  | Have any hand rinsates stayed overnight in the Hand Washing Facility container? | 1. Yes 2. No 3. Not applicable |
|  | Do all hand washing facilities have good drainage? | 1. Yes 2. No 3. Not applicable |
|  | Are there any splashes of wastewater from hand hygiene facilities in the healthcare facility? | 1. Yes 2. No 3. Not applicable |
|  | Are handwashing facilities at the healthcare facility clean? | 1. Yes 2. No 3. Not applicable |
|  | **Hand hygiene supplies at the healthcare facility** | |
|  | Does the healthcare facility have any alcohol based handrub in stock at the time of observation? (Ask to check the store) | 1. Yes 2. No 3. Not applicable |
|  | Does the healthcare facility have any soap in stock at the time of observation? (Ask to check the store) | 1. Yes 2. No 3. Not applicable |
|  | Is there evidence of stocktaking for hand hygiene facilities at the healthcare facility? | 1. Yes 2. No 3. Not applicable |
|  | **Comments:** *Enter anything you are concerned about that is not captured by*  *the questionnaire:* |  |
