# Supplementary material for: Impact of mhealth messages and environmental cues on hand hygiene practice among healthcare workers in the greater Kampala metropolitan area, Uganda: study protocol for a cluster randomized trial
Source: BMC Health Serv Res. 2021 Jan 26;21:88. doi: 10.1186/s12913-021-06082-3 (PMC7835669; doi:10.1186/s12913-021-06082-3)
Supplement: Supplementary file 5 — Additional file 5. [file 12913_2021_6082_MOESM5_ESM.docx]

## **Baseline, midline and end-line structured questionnaire for the HCWs**

| **Study Tittle:** Improving hand hygiene practice among healthcare workers through mhealth and environmental cues in Kampala Metropolitan Area.  **Preamble**  The interview with a healthcare provider should be conducted in as private a setting as you can find, and must be done individually. Data collectors should introduce themselves and explain the purpose of the survey saying that we are trying to find ways that our project can support the health services to improve hand hygiene in the healthcare facilities. You should inform the person that the interview will take about 15 minutes, and that the data you collect are confidential and that he/she will not be identified by name. Then request permission to conduct the interview.  Do not ask or write down the name of the person you are interviewing. If the person refuses to participate, accept the refusal and request to interview a different healthcare provider. If no one else is available or willing, report to your supervisor that the interview could not be completed at that facility. | | | |
| --- | --- | --- | --- |
| **IDENTIFICATION INFORMATION** | | | |
| **Date of interview (DD/MM/YYYY)** | |  | |
| **Unique ID** | |  | |
| **Initials of interviewer** | |  | |
| Name of the district | | 1. Kampala 2. Mukono 3. Wakiso | |
| Location of Health facility | | 1. Urban 2. Rural | |
| Name of healthcare facility | |  | |
| Level of Health Facility | | 1. Hospital 2. Health Centre IV 3. Health Centre III | |
| Department where Hand hygiene behavior observation | | 1. Maternity ward 2. Children’s ward 3. Other (specify) | |
| Ownership of Health Facility | | 1. Public 2. Private 3. PNFP | |
| Number of beds | |  | |
| Number of outpatients | |  | |
| Number of inpatients | |  | |
| Number of delivers per day | |  | |
| Number of healthcare workers | |  | |
| **SECTION 1: Socio demographic characteristics** | | | |
| **No.** | **Question** | **Response** | **Comment** |
|  | Sex | 1. Male 2. Female |  |
|  | Age (complete years) |  |  |
|  | Marital status | 1. Single (never married) 2. Married/cohabiting 3. Separated/divorced 4. Widowed |  |
|  | Profession | 1. Medical doctor 2. Clinical officer 3. Laboratory technician/ technologist 4. Laboratory assistant 5. Registered nurse 6. Enrolled nurse 7. Registered midwife 8. Enrolled midwife 9. Nursing assistant 10. Other (please specify) |  |
|  | Main department of work | 1. OPD 2. IPD 3. Maternity 4. Theatre 5. Laboratory 6. Others (specify) |  |
|  | For how long have you been practicing? (Duration in complete years) |  |  |
| **SECTION II: Knowledge on hand washing (Adapted from the WHO standardized questionnaire on hand hygiene)** | | | |
|  | Did you receive any formal training in hand hygiene in the last three years? | 1. Yes 2. No |  |
|  | Do you routinely use an alcohol-based hand rub for hand hygiene? | 1. Yes 2. No |  |
|  | Which of the following is the main route of cross-transmission of potentially harmful germs between patients in a health-care facility? (tick one answer only) | 1. Health-care workers’ hands when not clean 2. Air circulating in the hospital 3. Patients’ exposure to colonised surfaces (i.e., beds, chairs, tables, floors) 4. Sharing non-invasive objects (i.e., stethoscopes, pressure cuffs, etc.) between patients |  |
|  | What is the most frequent source of germs responsible for health care-associated infections?  (tick one answer only) | 1. The hospital’s water system 2. The hospital air 3. Germs already present on or within the patient 4. The hospital environment (surfaces) |  |
|  | Which of the following hand hygiene actions prevents transmission of germs to the patient? ***(tick all that apply)*** | \| **Actions** \| **Yes** \| **No** \| \| --- \| --- \| --- \| \| HW before touching a patient \|  \|  \| \| HW immediately after a risk of body fluid exposure \|  \|  \| \| HW after exposure to the immediate surroundings of a patient \|  \|  \| \| HW immediately before a clean/aseptic procedure \|  \|  \| |  |
|  | Which of the following hand hygiene actions prevents transmission of germs to the health-care worker? ***(tick all that apply)*** | \| **Actions** \| **Yes** \| **No** \| \| --- \| --- \| --- \| \| HW after touching a patient \|  \|  \| \| HW immediately after a risk of body fluid exposure \|  \|  \| \| HW after exposure to the immediate surroundings of a patient \|  \|  \| \| HW immediately before a clean/aseptic procedure \|  \|  \| \| HW after touching patient surroundings \|  \|  \| |  |
|  | Which of the following statements on alcohol-based hand rub and handwashing with soap and  water is true? | \| **Statement** \| **T** \| **F** \| \| --- \| --- \| --- \| \| Handrubbing is more rapid for hand cleansing than handwashing \|  \|  \| \| Handrubbing causes skin dryness more than handwashing \|  \|  \| \| Handrubbing is more effective against germs than handwashing \|  \|  \| \| Handwashing and handrubbing are recommended to be performed in sequence \|  \|  \| |  |
|  | What is the minimal time needed for alcohol-based handrub to kill most germs on your hands?  ***(tick one answer only)*** | 1. 20 seconds 2. 3 seconds 3. 1 minute 4. 10 seconds |  |
|  | Which type of hand hygiene method is required in the following situations?  **R=Rubbing, W=Washing, N=None** | \| **Situation** \| **R** \| **W** \| **N** \| \| --- \| --- \| --- \| --- \| \| Before palpation of the abdomen \|  \|  \|  \| \| Before giving an injection \|  \|  \|  \| \| After emptying a bedpan \|  \|  \|  \| \| After removing examination gloves \|  \|  \|  \| \| After making a patient's bed \|  \|  \|  \| \| After visible exposure to blood \|  \|  \|  \| | |
|  | Which of the following should be avoided, as associated with increased likelihood of colonisation of hands with harmful germs? **Y= Yes, N=No** | \| **Action to be avoided** \| **Y** \| **N** \| \| --- \| --- \| --- \| \| Wearing jewellery \|  \|  \| \| Damaged skin \|  \|  \| \| Artificial fingernails \|  \|  \| \| Regular use of a hand cream \|  \|  \| | |
| **SECTION III: PERCEPTION SURVEY FOR HEALTH-CARE WORKERS** | | | |
| **You are in direct contact with patients on a daily basis and this is why we are interested in your opinion on health care-associated infections and hand hygiene.** | | | |
|  | In your opinion, what is the average percentage of hospitalized patients who will develop a health care-associated infection (between 0 and 100%)? | 1. % 2. I don't know |  |
|  | In general, what is the impact of a health care-associated infection on a patient's clinical outcome? | 1. Very low 2. Low 3. High 4. Very high |  |
|  | What is the effectiveness of hand hygiene in preventing health care-associated infections? | 1. Very low 2. Low 3. High 4. Very high |  |
|  | Among all patient safety issues, how important is hand hygiene at your institution? | 1. Low priority 2. Moderate priority 3. High priority 4. Very high priority |  |
|  | On average, in what percentage of situations requiring hand hygiene do health-care workers in your hospital actually perform hand hygiene, either by hand-rubbing or handwashing (between 0 and 100%)? | 1. % 2. I don't know |  |
| **Opinions on the effectiveness of handwashing actions in a healthcare facility**  **In your opinion, how effective would the following actions be to improve hand hygiene permanently in your institution?** | | | |
|  | Leaders and senior managers at your institution support and openly promote hand hygiene. | 1. Very Frequently 2. Frequently 3. Occasionally 4. Rarely 5. Very Rarely 6. Never |  |
|  | The health-care facility makes alcohol-based handrub always available at each point of care. | 1. Very Frequently 2. Frequently 3. Occasionally 4. Rarely 5. Very Rarely 6. Never |  |
|  | Hand hygiene posters are displayed at point of care as reminders. | 1. Very Frequently 2. Frequently 3. Occasionally 4. Rarely 5. Very Rarely 6. Never |  |
|  | Each health-care worker receives education on hand hygiene | 1. Very Frequently 2. Frequently 3. Occasionally 4. Rarely 5. Very Rarely 6. Never |  |
|  | Each health-care worker receives text messages on hand hygiene | 1. Very Frequently 2. Frequently 3. Occasionally 4. Rarely 5. Very Rarely 6. Never |  |
|  | Clear and simple instructions for hand hygiene are made visible for every health-care worker | 1. Very Frequently 2. Frequently 3. Occasionally 4. Rarely 5. Very Rarely 6. Never |  |
|  | You always perform hand hygiene as recommended (being a good example for your colleagues). | 1. Very Frequently 2. Frequently 3. Occasionally 4. Rarely 5. Very Rarely 6. Never |  |
|  | Patients are invited to remind health-care workers to perform hand hygiene. | 1. Very Frequently 2. Frequently 3. Occasionally 4. Rarely 5. Very Rarely 6. Never |  |
| **SECTION IV: ATTITUDE TOWARDS HAND HYGIENE** | | | |
|  | Hand hygiene can be protective to HCWs | 1. Strongly agree 2. Agree 3. Indifferent 4. Disagree 5. Strongly disagree |  |
|  | Hand hygiene should be done when in contact with all patients and patient’s deformities | 1. Strongly agree 2. Agree 3. Indifferent 4. Disagree 5. Strongly disagree |  |
|  | I do not always adhere to hand hygiene because of a busy work schedule in between patients | 1. Strongly agree 2. Agree 3. Indifferent 4. Disagree 5. Strongly disagree |  |
|  | HCWs are motivated to do hand hygiene because of fear of contracting disease | 1. Strongly agree 2. Agree 3. Indifferent 4. Disagree 5. Strongly disagree |  |
|  | Hand hygiene can be improved by administrative order and continuous health education | 1. Strongly agree 2. Agree 3. Indifferent 4. Disagree 5. Strongly disagree |  |
|  | Hand hygiene in a healthcare setting is a waste of time | 1. Strongly agree 2. Agree 3. Indifferent 4. Disagree 5. Strongly disagree |  |
|  | Healthcare providers need reminders so as to practice hand hygiene | 1. Strongly agree 2. Agree 3. Indifferent 4. Disagree 5. Strongly disagree |  |
|  | Buying hand hygiene facilities and supplies in a healthcare facility is a waste of financial resources | 1. Strongly agree 2. Agree 3. Indifferent 4. Disagree 5. Strongly disagree |  |
|  | Hand hygiene should be a priority in all healthcare settings | 1. Strongly agree 2. Agree 3. Indifferent 4. Disagree 5. Strongly disagree |  |
|  | Hand hygiene should be compulsory in a healthcare setting | 1. Strongly agree 2. Agree 3. Indifferent 4. Disagree 5. Strongly disagree |  |
|  | Hand hygiene protects both the providers and patients from hospital acquired infections | 1. Strongly agree 2. Agree 3. Indifferent 4. Disagree 5. Strongly disagree |  |

| **SECTION V: HAND HYGIENE OBSERVATION SECTION** | | | | | |
| --- | --- | --- | --- | --- | --- |
|  | | **Period Number*****:** |  | **Session Number*****:** |  |
| **Service:** |  | **Date:  (dd/mm/yy)** | / / | **Observer:**  **(initials)** |  |
| **Ward:** |  | **Start/End time: (hh:mm)** | : / : | **Page N°:** |  |
| **Department:** |  | **Session duration: (mm)** |  | **District*****:** |  |

| **Prof.cat** | |  | | **Prof.cat** | |  | | **Prof.cat** | |  | | **Prof.cat** | |  | |
| --- | --- | --- | --- | --- | --- | --- | --- | --- | --- | --- | --- | --- | --- | --- | --- |
| **Code** | |  | | **Code** | |  | | **Code** | |  | | **Code** | |  | |
| **N°** | |  | | **N°** | |  | | **N°** | |  | | **N°** | |  | |
| **Opp.** | **Indication** | | **HH Action** | **Opp.** | **Indication** | | **HH Action** | **Opp.** | **Indication** | | **HH Action** | **Opp.** | **Indication** | | **HH Action** |
| **1** | bef-pat.  bef-asept.  aft-b.f.  aft-pat.  aft.p.surr. | | HR  HW  🌕 missed  🌕 gloves | **1** | bef-pat.  bef-asept.  aft-b.f.  aft-pat.  aft.p.surr. | | HR  HW  🌕 missed  🌕 gloves | **1** | bef-pat.  bef-asept.  aft-b.f.  aft-pat.  aft.p.surr. | | HR  HW  🌕 missed  🌕 gloves | **1** | bef-pat.  bef-asept.  aft-b.f.  aft-pat.  aft.p.surr. | | HR  HW  🌕 missed  🌕 gloves |
|  |  | |  |  |  | |  |  |  | |  |  |  | |  |
| **2** | bef-pat.  bef-asept.  aft-b.f.  aft-pat.  aft.p.surr. | | HR  HW  🌕 missed  🌕 gloves | **2** | bef-pat.  bef-asept.  aft-b.f.  aft-pat.  aft.p.surr. | | HR  HW  🌕 missed  🌕 gloves | **2** | bef-pat.  bef-asept.  aft-b.f.  aft-pat.  aft.p.surr. | | HR  HW  🌕 missed  🌕 gloves | **2** | bef-pat.  bef-asept.  aft-b.f.  aft-pat.  aft.p.surr. | | HR  HW  🌕 missed  🌕 gloves |
|  |  | |  |  |  | |  |  |  | |  |  |  | |  |
| **3** | bef-pat.  bef-asept.  aft-b.f.  aft-pat.  aft.p.surr. | | HR  HW  🌕 missed  🌕 gloves | **3** | bef-pat.  bef-asept.  aft-b.f.  aft-pat.  aft.p.surr. | | HR  HW  🌕 missed  🌕 gloves | **3** | bef-pat.  bef-asept.  aft-b.f.  aft-pat.  aft.p.surr. | | HR  HW  🌕 missed  🌕 gloves | **3** | bef-pat.  bef-asept.  aft-b.f.  aft-pat.  aft.p.surr. | | HR  HW  🌕 missed  🌕 gloves |
|  |  | |  |  |  | |  |  |  | |  |  |  | |  |
| **4** | bef-pat.  bef-asept.  aft-b.f.  aft-pat.  aft.p.surr. | | HR  HW  🌕 missed  🌕 gloves | **4** | bef-pat.  bef-asept.  aft-b.f.  aft-pat.  aft.p.surr. | | HR  HW  🌕 missed  🌕 gloves | **4** | bef-pat.  bef-asept.  aft-b.f.  aft-pat.  aft.p.surr. | | HR  HW  🌕 missed  🌕 gloves | **4** | bef-pat.  bef-asept.  aft-b.f.  aft-pat.  aft.p.surr. | | HR  HW  🌕 missed  🌕 gloves |

General Recommendations

* To be completed by the data manager.

** **Optional**, to be used if appropriate, according to the local needs and regulations.

(refer to the Hand Hygiene Technical Reference Manual)

1. In the context of open and direct observations, the observer introduces him/herself to the health-care worker and to the patient when appropriate, explains his/her task and proposes immediate informal feedback.
2. The health-care worker, belonging to one of the main four following professional categories (see below), is observed during the delivery of health-care activities to patients.
3. Detected and observed data should be recorded with a pencil in order to be immediately corrected if needed.
4. The top of the form (header) is completed before starting data collection (excepted end time and session duration).
5. The session should last no more than 20 minutes (± 10 minutes according to the observed activity); the end time and the session duration are to be completed at the end of the observation session.
6. The observer may observe up to three health-care workers simultaneously, if the density of hand hygiene opportunities permits.
7. Each column of the grid to record hand hygiene practices is intended to be dedicated to a specific professional category. Therefore, numerous health-care workers may be sequentially included during one session in the column dedicated to their category. Alternatively, each column may be dedicated to a single health-care worker only of whom the professional category should be indicated.
8. As soon as you detect an indication for hand hygiene, count an opportunity in the appropriate column and cross the square corresponding to the indication(s) you detected. Then complete all the indications that apply and the related hand hygiene actions observed or missed.
9. Each opportunity refers to one line in each column; each line is independent from one column to another.
10. Cross items in squares (several may apply for one opportunity) or circles (only a single item may apply at one moment).
11. When several indications fall in one opportunity, each one must be recorded by crossing the squares.
12. Performed or missed actions must always be registered within the context of an opportunity.
13. Glove use may be recorded only when the hand hygiene action is missed while the health-care worker is wearing gloves.

Short description of items

| **Facility:** | to complete according to the local nomenclature | | |
| --- | --- | --- | --- |
| **Service:** | to complete according to the local nomenclature | | |
| **Ward:** | to complete according to the local nomenclature | | |
| **Department:** | to complete according to the following standardized nomenclature: | | |
|  | medical, including dermatology, neurology, haematology, oncology, etc. | | surgery, including neurosurgery, urology, EENT, ophthalmology, etc. |
|  | mixed (medical & surgical), including gynaecology | | obstetrics, including related surgery |
|  | paediatrics, including related surgery | | intensive care & resuscitation |
|  | emergency unit | | long term care & rehabilitation |
|  | ambulatory care, including related surgery | | other (to specify) |
| **Period N°:** | 1) pre- / 2) post-intervention; and then according to the institutional counter. | | |
| **Date:** | day (dd) / month (mm) / year (yy) | | |
| **Start/end time:** | hour (hh) / minute (mm). | | |
| **Session duration:** | difference between start and end time, resulting in minutes of observation. | | |
| **Session N°:** | attributed at the moment of data entry for analysis. | | |
| **Observer:** | observer’s initials (the observer is responsible for the data collection and for checking their accuracy before submitting the form for analysis. | | |
| **Page N°:** | to write only when more than one form is used for one session. | | |
| **Prof.cat:** | according to the following classification: | | |
|  | **1. nurse / midwife** | 1.1 nurse, 1.2 midwife, 1.3 student. | |
|  | **2. auxiliary** |  | |
|  | **3. medical doctor** | 3.1 in internal medicine, 3.2 surgeon, 3.3 anaesthetist / resuscitator / emergency physician, 3.4 paediatrician, 3.5 gynaecologist, 3.6 consultant, 3.7 medical student. | |
|  | **4. other health-care worker** | 4.1 therapist (physiotherapist, occupational therapist, audiologist, speech therapist), 4.2 technician (radiologist, cardiology technician, operating room technician, laboratory technician, etc), 4.3 other (dietician, dentist, social worker and any other health-related professional involved in patient care), 4.4 student. | |
| **Number:** | number of observed health-care workers belonging to the same professional category (same code) as they enter the field of observation and you detect opportunities. | | |
| **Opp(ortunity):** | defined by one indication at least | | |
| **Indication:** | reason(s) that motivate(s) hand hygiene action; all indications that apply at one moment must be recorded | | |
|  | bef.pat: before touching a patient | | aft.b.f: after body fluid exposure risk |
|  | bef.asept: before clean/aseptic procedure | | aft.pat: after touching a patient |
|  |  | | aft.p.surr: after touching patient surroundings |
| **HH action:** | response to the hand hygiene indication(s); it can be either a positive action by performing handrub or handwash, or a negative action by missing handrub or handwash | | |
|  | HR: hand hygiene action by handrubbing with an alcohol-based formula  HW: hand hygiene action by handwashing with soap and water | | Missed: no hand hygiene action performed |

Observation Form – Basic Compliance Calculation

draft

|  | **Facility:** | | | | | | | | **Period:** | | | | **Setting:** | | | | | |
| --- | --- | --- | --- | --- | --- | --- | --- | --- | --- | --- | --- | --- | --- | --- | --- | --- | --- | --- |
|  | **Prof.cat.** | | | | **Prof.cat.** | | | | **Prof.cat.** | | | | **Prof.cat.** | | | **Total per session** | | |
| **Session N°** | **Opp (n)** | **HW (n)** | **HR**  **(n)** | **Opp (n)** | | **HW (n)** | **HR**  **(n)** | **Opp (n)** | | **HW (n)** | **HR**  **(n)** | **Opp (n)** | | **HW (n)** | **HR**  **(n)** | **Opp (n)** | **HW (n)** | **HR**  **(n)** |
| **1** |  |  |  |  | |  |  |  | |  |  |  | |  |  |  |  |  |
| **2** |  |  |  |  | |  |  |  | |  |  |  | |  |  |  |  |  |
| **3** |  |  |  |  | |  |  |  | |  |  |  | |  |  |  |  |  |
| **4** |  |  |  |  | |  |  |  | |  |  |  | |  |  |  |  |  |
| **5** |  |  |  |  | |  |  |  | |  |  |  | |  |  |  |  |  |
| **6** |  |  |  |  | |  |  |  | |  |  |  | |  |  |  |  |  |
| **7** |  |  |  |  | |  |  |  | |  |  |  | |  |  |  |  |  |
| **8** |  |  |  |  | |  |  |  | |  |  |  | |  |  |  |  |  |
| **9** |  |  |  |  | |  |  |  | |  |  |  | |  |  |  |  |  |
| **10** |  |  |  |  | |  |  |  | |  |  |  | |  |  |  |  |  |
| **11** |  |  |  |  | |  |  |  | |  |  |  | |  |  |  |  |  |
| **12** |  |  |  |  | |  |  |  | |  |  |  | |  |  |  |  |  |
| **13** |  |  |  |  | |  |  |  | |  |  |  | |  |  |  |  |  |
| **14** |  |  |  |  | |  |  |  | |  |  |  | |  |  |  |  |  |
| **15** |  |  |  |  | |  |  |  | |  |  |  | |  |  |  |  |  |
| **16** |  |  |  |  | |  |  |  | |  |  |  | |  |  |  |  |  |
| **17** |  |  |  |  | |  |  |  | |  |  |  | |  |  |  |  |  |
| **18** |  |  |  |  | |  |  |  | |  |  |  | |  |  |  |  |  |
| **19** |  |  |  |  | |  |  |  | |  |  |  | |  |  |  |  |  |
| **20** |  |  |  |  | |  |  |  | |  |  |  | |  |  |  |  |  |
| **Total** |  |  |  |  | |  |  |  | |  |  |  | |  |  |  |  |  |
| **Calculation** | **Act (n) =**  **Opp (n) =** | | | **Act (n) =**  **Opp (n)** = | | | | | **Act (n) =**  **Opp (n) =** | | | **Act (n)** =  **Opp (n) =** | | | | **Act (n)** =  **Opp (n) =** | | |
| **Compliance** |  | | |  | | | | |  | | |  | | | |  | | |

Compliance (%) = Actions x 100

Opportunities

Instructions for use

1. Define the setting outlining the scope for analysis and report related data according to the chosen setting.
2. Check data in the observation form. Hand hygiene actions not related to an indication should not be taken into account and vice versa.
3. Report the session number and the related observation data in the same line. This attribution of session number validates the fact that data has been taken into count for compliance calculation.
4. Results per professional category and per session (vertical):

4.1 Sum up recorded opportunities (opp) in the case report form per professional category: report the sum in the corresponding cell in the calculation form.

4.2 Sum up the positive hand hygiene actions related to the total of opportunities above, making difference between handwash (HW) and handrub (HR): report the sum in the corresponding cell in the calculation form.

4.3 Proceed in the same way for each session (data record form).

4.4 Add up all sums per each professional category and put the calculation to calculate the compliance rate (given in percent)

1. The addition of results of each line permits to get the global compliance at the end of the last right column.
