# Supplementary material for: Impact of mhealth messages and environmental cues on hand hygiene practice among healthcare workers in the greater Kampala metropolitan area, Uganda: study protocol for a cluster randomized trial
Source: BMC Health Serv Res. 2021 Jan 26;21:88. doi: 10.1186/s12913-021-06082-3 (PMC7835669; doi:10.1186/s12913-021-06082-3)
Supplement: Supplementary file 6 — Additional file 6. [file 12913_2021_6082_MOESM6_ESM.docx]

**INFORMED WRITTEN CONSENT FORM FOR HEALTHCARE WORKERS**

**Project tittle:** The impact of an mhealth and environmental cues intervention on hand hygiene practice among healthcare workers in the Greater Kampala Metropolitan Area: a cluster randomised trial.

**Study Investigators:** Richard K. Mugambe, Ntanda Moses, Semwanga David, Tonny Ssekamatte, Winnie Kansiime.

**Introduction**

Hello, my name is _______________________. I work with Makerere University School of Public Health. I would like to invite you to participate in a cluster randomised trial on improving hand hygiene practice among healthcare workers through mhealth and environmental cues. The cluster randomised trial is called “The impact of an mhealth and environmental cues intervention on hand hygiene practice among healthcare workers in the Greater Kampala Metropolitan Area.”

**Objective of the study/trial**

The objective of the trial is to determine the impact of mobile phone WASH text messages and environmental clues on hand hygiene practice among healthcare workers in the Greater Kampala Metropolitan Area. Specifically, the trial aims at 1) exploring the opportunities for enhancing healthcare workers’ hand hygiene practices in HCFs in the GKMA, 2) exploring the barriers for enhancing healthcare workers’ hand hygiene practices in HCFs and 3) determine the impact of mhealth messages and environmental cues on hand hygiene practice among health workers in HCFs in the GKMA.

**Trial/ study procedure**

The trial will be implemented following five stages. 1) **Stage 1**: The Assess stage will involve document existing hand hygiene behaviour 2) **Stage 2**: Build - Formative phase: This will involve conducting key informant and in-depth interviews using a key informant interview guide and an in-depth interview guide respectively. In addition, we shall conduct observations in 100 healthcare facilities using a structured observation checklist to establish the status of infrastructure and behaviour settings. Data obtained in stage 1 and 2 will inform the **Stage 3:** Create stage: During the create stage, we shall share findings with the stakeholders on the barriers and facilitators of hand hygiene and discuss contextual mechanisms of designing and implementing the intervention (mhealth and environmental cues). Afterwards, we shall implement **Stage 4-** Delivery. This stage will involve the randomisation of healthcare providers in to the intervention and control healthcare facilities. In both the intervention and control healthcare facilities, simple hand washing facilities (HWF) (tapped plastic jerricans with a stand and basin) will be provided in delivery rooms, post-natal wards, and children’s ward. Soap for hand washing as well as alcohol hand rub will also be provided at the hand hygiene stations. In the intervention HCFs, besides the provisions in the control group, two interventions, mhealth and environmental cues (nudges) will be provided. The choice of the specific mhealth intervention will be determined in the insight workshops, and participants will be exposed to messages three times a week for a period of 4 months. The mhealth intervention messages will be designed with a focus on: information on the benefits of hand hygiene; when to do hand hygiene; how to do hand hygiene and how to protect others. After the delivery of the intervention, we shall undertake S**tage 5-**Evaluation which will involve the evaluation of the intervention to assess its impact on hand hygiene practice.

**Risks from Being in the Study**

Potential risks include:

- The risk that your views may become known to other people who have not participated in the interviews. We will minimize this by ensuring that only the individual directly responsible for this study will have access to the interviews. Only authorized project personnel (approved by the study Principal Investigator) will have access to this information.

**Benefits**

- An opportunity to have your ideas shared with policy makers and program implementers to influence and contribute to the promotion of hand hygiene practice in healthcare facilities.

**Assurance of Confidentiality**

Information collected from you will be kept confidential (secret) by Makerere University of Public Health to the full extent allowed by law. All data will be kept under password-protected computers to avoid unauthorized access to the data. Finally, your name will not be linked to your views; we will report about people’s views in general and no attempt will be made to link the views to those who shared them.

**Participation is Voluntary**

This interview will take approximately 45 minutes. Your participation in this study is strictly voluntary and you do not have to participate if you do not want to or to answer every question if you don’t feel comfortable. Your responses will be entered into a mobile phone application and later uploaded to a password protected online platform. You may request that the interview be paused at any time. You can also withdraw from this study at any time without any reprisal.

**Compensation**  The study is on voluntary basis and no monetary benefits will be accrued by the study participants.

**Questions/Points of Contact**

If you have any questions for me, about the study or the consent document, please ask before signing, and I will do my best to answer them. You will receive a copy of this consent form. If you have additional questions or if you need to discuss any other aspect of the study, you can contact: the Principal Investigator; Dr. Richard Mugambe, Makerere University School of Public Health P. O. Box 7072, Kampala, (+256 789 477 677) or by email on, [rmugambe@musph.ac.ug](mailto:rmugambe@musph.ac.ug).

This study has been reviewed and approved by the Makerere University School of Public Health Higher Degrees, Research and Ethics Committee and by the Uganda National Council for Science and Technology. If you have any questions concerning your rights as a participant in this research, please contact Dr Suzanne Kiwanuka, Chairperson of the Higher Degrees, Research and Ethics Committee at Makerere University School of Public Health (tel. 0772 886377).

**Statement of Participant Consent**

I have been asked to participate in a research study named “The impact of an mhealth and environmental cues intervention on hand hygiene practice among healthcare workers in the Greater Kampala Metropolitan Area. A Cluster Randomised Trial.” The principal investigator, Dr. Richard K. Mugambe, or his representative, ___________________, has explained the study to me and the risks this might involve. The information was read to me and I have been given an opportunity to ask questions. All questions were answered in a way that I understand. If I have other questions about this research, I can ask the study representative, ________________, or contact Dr. Richard K. Mugambe. I understand that my agreement to participate in this study is voluntary, and that I can decline to participate or leave the study at any time. I also understand that I have the right to voluntarily refuse to participate in all or part of the study. I am signing my name below to indicate my consent to participate in this study. I will be given a copy of the signed consent form.

___________________ __________________________ ______________________

Signature of participant Printed name of participant Date

___________________ ________________________ ________________________

Signature of witness Printed name of witness Date

____________________ _________________________ ________________________

Signature of investigator Printed name of witness Date

eliciting consent
